# Circulating succinate changes during acute cold exposure are not related with brown adipose tissue in humans

**DOI:** 10.1007/s13105-026-01212-z

**Published:** 2026-08-01

**Authors:** Francisco J. Osuna-Prieto, Borja Martinez-Tellez, Guillermo Sánchez-Delgado, Francisco M. Acosta, Lucas Jurado-Fasoli, Joan Vendrell, Jonatan R. Ruiz, Sonia Fernández-Veledo

**Affiliations:** 1 DIAMET Research Group, Institut de Recerca Biomèdica Catalunya Sud (IRB CatSud), Tarragona, Spain; 2 CIBER de Diabetes y Enfermedades Metabólicas Asociadas (CIBERDEM)-Instituto de Salud Carlos III (ISCIII), Madrid, 28029 Spain; 3 Department of Nursing, Physiotherapy and Medicine and SPORT Research Group, CIBIS Research Center, University of Almería, Almería, 04120 Spain; 4 Biomedical Research Unit, Torrecárdenas University Hospital, Almería, 04009 Spain; 5 CIBER de Fisiopatología de la Obesidad y Nutrición (CIBEROBN), Instituto de Salud Carlos III, Granada, 18012 Spain; 6 Department of Medicine, Division of Endocrinology and Einthoven Laboratory for Experimental Vascular Medicine, Leiden University Medical Center, Leiden, The Netherlands; 7 Department of Physiology, Faculty of Medicine, Sport and Health University Research Institute (iMUDS), University of Granada, Granada, Andalucía Spain; 8 Instituto de Investigación Biosanitaria (Ibs), Granada, 18014 Spain; 9 Turku PET Centre, University of Turku, Turku, Finland; 10 Turku PET Centre, Turku University Hospital, Turku, Finland; 11 InFLAMES Research Flagship Center, University of Turku, Turku, Finland; 12 MediCity Research Laboratories, University of Turku, Turku, Finland; 13 Universitat Rovira i Virgili (URV), Reus, 43201 Spain; 14 Department of Physical Education and Sports, Faculty of Sports Science, Sport and Health, University Research Institute (iMUDS), University of Granada, Crta Alfcar s/n. 18011, Granada, 18071 Spain

**Keywords:** Brown fat, Thermogenesis, Cold-induced thermogenesis, Uncoupling-protein 1 (UCP1)

## Abstract

Brown adipose tissue (BAT) activity in humans is linked to better glucose metabolism and cardiometabolic health. In mice, cold exposure increases circulating succinate and enhances BAT thermogenesis, but whether similar succinate responses occur in humans remains undetermined. We analysed data from 33 young (18–25 years), sedentary adults (20 women) from the ACTIBATE cohort (NCT02365129). Plasma succinate was measured enzymatically at baseline and 60 and 120 min during a personalized cooling protocol. BAT volume, activity, and radiodensity were assessed via static ^18^F-fluorodeoxyglucose positron emission tomography–computed tomography (^18^F-FDG PET-CT) at the end of the cold exposure. Circulating succinate changed over time during cold exposure, with no significant change at 60 min and a significant increase at 120 min versus baseline (+ 23.8%, *P* = 0.017), with substantial interindividual variability. Exploratory stratification by median baseline succinate revealed divergent temporal patterns: the low-baseline group showed increases at 60 min (+ 46%) and 120 min (+ 80.5%; both *P* ≤ 0.021), while the high-baseline group showed reductions at both timepoints (–31.4% and − 2.4%; both *P* ≤ 0.003). These profiles were not explained by phenotypic or BAT-related parameters. In the full cohort, changes in circulating succinate were not associated with BAT outcomes. Overall, our findings indicate that circulating succinate responses during acute cold exposure are highly heterogeneous and no associated with BAT PET/CT-derived parameters in humans.

**Supplementary Information:**

The online version contains supplementary material available at 10.1007/s13105-026-01212-z.

## Introduction

Brown adipose tissue (BAT) is a specialized fat depot that regulates body temperature through non-shivering thermogenesis, a process that generates heat in response to cold exposure and β-adrenergic stimulation [1]. This process is mainly driven by uncoupling protein 1 (UCP1), which facilitates heat production by uncoupling oxidative phosphorylation in brown adipocyte mitochondria [2]. However, UCP1-independent thermogenic mechanisms have also been described [3, 4]. BAT has garnered significant attention for its attributed metabolic benefits, as preclinical studies show it can counteract obesity and cardiometabolic disorders by increasing energy expenditure and improving metabolic homeostasis [5]. In humans, active BAT is associated with better glucose metabolism and insulin sensitivity [6], with studies linking higher BAT activity to a healthier cardiometabolic profile, even in individuals with severe obesity [7, 8].

Given BAT’s therapeutic potential against obesity and metabolic diseases, substantial efforts have been directed toward developing pharmacological agents that stimulate BAT thermogenesis. Most strategies have focused on targeting β-adrenergic signalling pathways [9–11]; however, despite promising results in rodent models, many interventions have failed to translate effectively to humans, and no such therapies have yet reached clinical application for the treatment of obesity or type 2 diabetes [12]. In preclinical models, succinate (a key intermediate of the tricarboxylic acid (TCA) cycle) has emerged as a activator of BAT thermogenesis, even in the absence of adrenergic input [13]. In mice, cold exposure increases circulating succinate levels, which have been proposed to be taken up by brown adipocytes adipocytes in murine models, potentially contributing to UCP1-dependent thermogenesis [13, 14]. This uptake occurs via monocarboxylate transporters (MCTs), supporting a thermogenic mechanism that is independent of succinate receptor 1 (SUCNR1)-mediated signalling [14]. In this regard, skeletal muscle has been proposed as a source of circulating succinate during early or low-intensity cold-induced shivering, potentially supporting BAT activation [15, 16]. Nevertheless, given succinate’s central role in mitochondrial metabolism, other metabolically active tissues may also contribute to its systemic rise during cold exposure [17].

Although studies in mice demonstrate that cold-induced increases in circulating succinate promote BAT thermogenesis, it remains unclear whether similar responses occur in humans. In this study, we examined the effects of a 2-h individualized cold exposure protocol on plasma succinate levels and their associations with BAT volume, ^18^F-fluorodeoxyglucose (^18^F-FDG) uptake, and radiodensity in humans.

## Methods

### Participants

These analyses were conducted using baseline data from the ACTIBATE study, which aimed to assess the effects of concurrent exercise training on BAT volume, activity, and radiodensity (ClinicalTrials.gov ID: NCT02365129; clinical registration date 2015-02−10) [18]. We included 33 young healthy adults (20 women), as shown in Table 1. Participants were recruited through electronic media advertisements and distributed leaflets. Before enrolment, all individuals provided written informed consent. Eligibility criteria included being aged 18 to 25 years, a self-reported sedentary lifestyle (defined as engaging in less than 20 min of moderate to vigorous physical activity on fewer than three days per week, self-reported), non-smoking, having a stable body weight (with no changes exceeding 3 kg over the last three months), and being free from cardiometabolic diseases (e.g., hypertension or diabetes). Participants were also excluded if taking medications that affect cardiovascular function or had a cancer antecedent in first-degree relatives.

**Table 1:** Characteristics of the participants included in the study

	All (*n* = 33)		Men (*n* = 13)		Women (*n* = 20)		*P* sex
	Mean	SD	Mean	SD	Mean	SD	
Age (years)	21.8	2.2	22.5	2.3	21.4	2.1	0.162
*Body composition*							
BMI (kg/m^2^)	24.0	5.3	26.5	6.7	22.4	3.6	0.059
Lean mass index (kg/m^2^)	14.6	2.4	16.5	2.2	13.3	1.5	**< 0.001**
Fat mass index (kg/m^2^)	8.0	3.4	8.4	4.5	7.8	2.4	0.605
Fat mass (%)	33.2	7.7	30.7	9.7	34.9	5.7	0.124
VAT mass (g)	303.0	197.5	429.4	220.2	220.8	130.4	**0.002**
*Cardiometabolic risk factors*							
Glucose (mg/dL)	87.7	7.4	90.7	8.5	85.7	5.9	0.057
Insulin (µIU/mL)	9.2	8.0	11.3	11.8	7.8	3.6	0.225
HOMA-IR	2.1	2.2	2.7	3.3	1.7	0.8	0.198
Total cholesterol (mg/dL)	163.8	35.4	161.8	46.0	165.2	27.3	0.792
HDL-C (mg/dL)	51.4	11.0	45.3	7.4	55.6	11.3	**0.007**
LDL-C (mg/dL)	95.7	26.6	97.4	32.8	94.5	22.3	0.767
Triglycerides (mg/dL)	86.2	56.2	101.4	82.0	75.8	25.9	0.211
APOA1 (mg/dL)	143.9	26.2	124.8	22.0	154.1	22.7	0.007
APOB (mg/dL)	64.1	15.1	64.5	12.8	63.9	16.7	0.926
Adiponectin (mg/L)	11.9	8.9	8.7	7.2	13.9	9.4	0.119
Leptin (µg/L)	4.4	2.8	3.3	3.0	5.1	2.6	0.086
GPT (IU/L)	22.2	24.7	33.2	35.5	14.7	8.1	0.089
GGT (IU/L)	23.5	27.0	37.7	38.6	13.8	4.1	**0.028**
ALP (IU/L)	73.1	24.3	85.6	28.7	64.5	16.5	**0.013**
Creatinine (mg/dL)	0.8	0.1	0.9	0.2	0.8	0.1	**0.003**
Creatine kinase (µmol/L)	126.9	134.5	189.4	194.5	84.2	34.6	0.223
C-reactive protein (mg/L)	2.4	2.8	3.1	3.3	1.9	2.3	0.077
*Physical fitness*							
Handgrip strength (kg)	31.1	8.2	38.5	7.7	26.2	3.7	**< 0.001**
RM bench press (kg)	33.4	15.6	47.9	14.7	24.0	6.1	**< 0.001**
RM leg press (kg)	210.0	72.8	277.4	55.0	166.2	43.5	**< 0.001**
VO_2_peak (mL/kg/min)	42.7	8.2	43.7	11.3	42.0	5.9	0.587
*Brown adipose tissue*							
BAT volume (mL)	58.1	73.2	74.4	157.4	54.1	75.5	0.353
BAT SUVmean	4.0	2.2	4.0	2.2	3.9	2.3	0.742
BAT SUVpeak	11.9	11.8	12.7	13.2	10.2	11.6	0.531
BAT radiodensity (HU)	−58.4	13.1	−58.8	14.7	−58.1	12.8	0.984

Data presented as mean and standard deviation (SD). BAT radiodensity sample size: *Abbreviations*: ALP, alkaline phosphatase; APOA1, apolipoprotein A1; APOB, apolipoprotein B; BAT, brown adipose tissue; BMI, body mass index; GGT, gamma-glutamyl transferase; GPP, glutamic pyruvic transaminase; HDL-C, high-density lipoprotein cholesterol; HOMA-IR, homeostatic model assessment of insulin resistance index; HU, Hounsfield Units; LDL-C, low-density lipoprotein cholesterol; RM: repetition maximum; SUV, standardized uptake value; VAT, visceral adipose tissue: VO_2_: oxygen consumption

## Compliance with ethical standards

### Research involving human participants and/or animals

This study involved human participants and did not include any animal experiments. All procedures were conducted in accordance with the ethical standards of the institutional and/or national research committees and with the Declaration of Helsinki (2013).

### Ethics approval declaration

Ethical approval was obtained from the Human Research Ethics Committee of the University of Granada (approval number 924) and the Servicio Andaluz de Salud.

### Informed consent

Freely-given written informed consent to participate in the study was obtained from all participants prior to enrolment and before any study-related procedures.

### Consent to publish

All participants provided written informed consent for the publication of anonymized data.

### Disclosure of potential conflicts of interest

The authors declare that they have no conflicts of interest.

### Blood sample collection and succinate determination

Before cold exposure, participants completed at least 6 h of fasting prior to the protocol to minimize acute postprandial increases in circulating metabolites that could influence BAT activation and whole-body energy expenditure in humans [18, 19], including succinate [20]. An intravenous catheter was inserted into the antecubital vein, and blood samples were drawn at baseline and 60 and 120 min during the cooling protocol. Additional blood samples to assess cardiometabolic risk factors were obtained on a separate day between 8:00 and 11:00 a.m. following a 10-h overnight fast. Blood samples were immediately processed by centrifugation to separate serum (collected using Vacutainer^®^ SST™ II Advance tubes) and plasma (collected using Vacutainer^®^ Hemogard™ tubes containing EDTA as an anticoagulant). The aliquoted serum and plasma samples were stored at −80 °C for future analysis.

Plasma succinate concentrations were determined using the EnzyChrom™ Succinate Assay Kit (BioAssay Systems, Hayward, CA). A detailed description of how cardiometabolic risk factors and physical fitness parameters were determined can be found in the Supplementary Material.

### Personalized cooling protocol and brown adipose tissue assessment

To individualize the cooling protocol used for BAT activation, each participant’s shivering threshold was determined 48–72 h before the assessment of BAT volume and metabolic activity. In brief, participants initially spent 30 min in a warm room (22.1 ± 1.6 °C) to acclimate, after which they were moved to a mild-cold (19.8 ± 0.5 °C) room. During this time, participants wore a cooling vest (Polar Products Inc., Stow, OH, USA), with water temperature set at 16.6 °C and gradually reduced every 10 min until reaching 5.5 °C. If no shivering (i.e., the moment at which shivering was visually observed or reported by the participant) was observed at that point, the temperature was further decreased by 0.6 °C every 15 min down to a minimum of 3.8 °C. In cases where shivering still did not occur, participants remained in the cold room for an additional 45 min with the water maintained at 3.8 °C.

After a 48–72-h interval, participants returned to the laboratory for a second round of cooling, during which they were exposed to a 2-h cold protocol at approximately 4 °C above their shivering threshold. Following 1 h of cold exposure, a bolus of approximately 185 MBq of ^1818^F-FDGnwas administered intravenously, and a positron emission tomography/computed tomography (PET/CT) scan was performed two h later (Siemens Biograph 16 PET/CT, Siemens Healthcare, Erlangen, Germany). PET/CT images were analysed using the open-source Beth Israel plugin for the FIJI program [21]. To quantify BAT volume and activity, an individualized SUV threshold for a voxel to be considered to represent BAT was taken as ≥ [1.2/(lean body mass/body mass) [22], using a Hounsfield Unit range from − 190 to −10. BAT volume was determined by quantifying pixels in this range with an SUV above the threshold. Multiple regions of interest (ROIs) were semiautomatically defined on both sides of the body, spanning from the atlas to the fourth thoracic vertebra. These included the laterocervical, supraclavicular, mediastinal, and paravertebral regions. Activity (^18^F-FDG uptake) was assessed by calculating the mean SUV (SUVmean) in the same pixels, reflecting the average quantity of ^18^F-FDG uptake in BAT, and the peak SUV (SUVpeak), which was the highest SUV value within a 1 cm³ volume. BAT mean radiodensity was also quantified. This measurement primarily reflects tissue composition in terms of relative lipid (i.e., triglyceride) and water content, since lipids exhibit low negative HU values (typically between − 190 and − 30 HU in adipose tissue), whereas water and other non-lipid components have higher around 0 values [23, 24]. In the context of BAT, radiodensity after cold exposure is as an indirect marker of intracellular triglyceride content, where higher radiodensity (i.e., less negative or closer to 0 HU) could be interpreted as a reduction in lipid content due to mobilization and oxidation of fatty acids during thermogenic activation [23, 24].

### Anthropometry and body composition

Waist circumference was measured at the minimum using a measuring tape with 1-mm precision, taken at the end of a normal exhalation, with the participant’s arms relaxed by their sides. If the narrowest point was not identifiable (e.g., in individuals with higher body mass), measurements were taken 2 cm above the umbilicus, following a horizontal plane. Body weight and height were assessed using a SECA model 799 electronic column scale and a stadiometer (SECA, Hamburg, Germany).

Lean body mass (LBM), fat mass, and visceral adipose tissue (VAT) mass were determined using a Hologic Discovery Wi dual-energy X-ray absorptiometer (DXA) (Hologic, Marlborough, MA, USA). Body mass index (BMI), lean mass index (LMI), and fat mass index (FMI) were computed by dividing body weight, LBM, and fat mass (in kg) by the square of height (in cm).

### Statistical analysis

Data were tested for normality using both the Shapiro–Wilk and Kolmogorov–Smirnov tests, alongside visual inspection of frequency histograms and Q–Q plots. Normally distributed variables are presented as means ± standard deviations, while non-normally distributed variables are reported as medians and interquartile ranges (IQR). Cold-induced changes in circulating succinate concentrations were analyzed using linear mixed-effects models with restricted maximum likelihood estimation (REML). Time (baseline, 60 min, 120 min) was included as a fixed within-subject factor, and subject ID was modeled as a random intercept to account for repeated measures. The covariance structure for repeated measures was specified as compound symmetry. Degrees of freedom were estimated using the Satterthwaite method. Main effects were assessed using Type III sums of squares, and post-hoc comparisons of adjusted means were performed with Bonferroni correction. To explore potential modulators of the succinate response, we repeated these analyses introducing sex, BMI, LBM, and VAT mass as fixed factors (BMI, LBM, and VAT mass computed as categorical variables based on median levels), testing for *Time x Group* interactions. A similar approach was employed to study the succinate response between low and high-baseline succinate groups, including group (low or high-baseline succinate) as a fixed factor. This exploratory post-hoc stratification by median baseline succinate was used solely to visualize interindividual heterogeneity. To identify predictors of Δ succinate at 60 min and 120 min, stepwise linear regression models were performed using a comprehensive panel of phenotypic variables as independent predictors, including body composition, cardiometabolic markers, physical fitness, and BAT-related parameters. To minimize the risk of overfitting given the sample size, a maximum of three variables per category were included in each model. To avoid limitations inherent to delta-based analyses, we also assessed whether baseline succinate concentrations predicted absolute values at 60 min and 120 min. Linear regression analyses were then used to test associations between changes in plasma succinate (Δ succinate 60 and Δ succinate 120 min) and BAT-related outcomes (volume, ^18^F-FDG uptake, and radiodensity). These analyses were performed both in the full cohort and then stratified by median baseline succinate levels (low vs. high). Differences between phenotypic traits between independent groups (e.g., male and females; low vs. high baseline succinate groups) were evaluated using independent-samples *t* tests. All models were adjusted separately for potential confounders, including cooling vest temperature, body surface area, and the PET-CT scan date and time of day. All analyses were conducted using SPSS (v29, IBM Corp.) and figures built with Prism (v8, GraphPad), with statistical significance set at *P* < 0.05.

### Role of funders

Funders played no role in study design, data acquisition, analysis, or manuscript drafting.

## Results

The characteristics of the participants of the study are summarized in Table 1.

### Changes in plasma succinate levels during cold exposure are not associated with BAT parametes

**Fig. 1:**
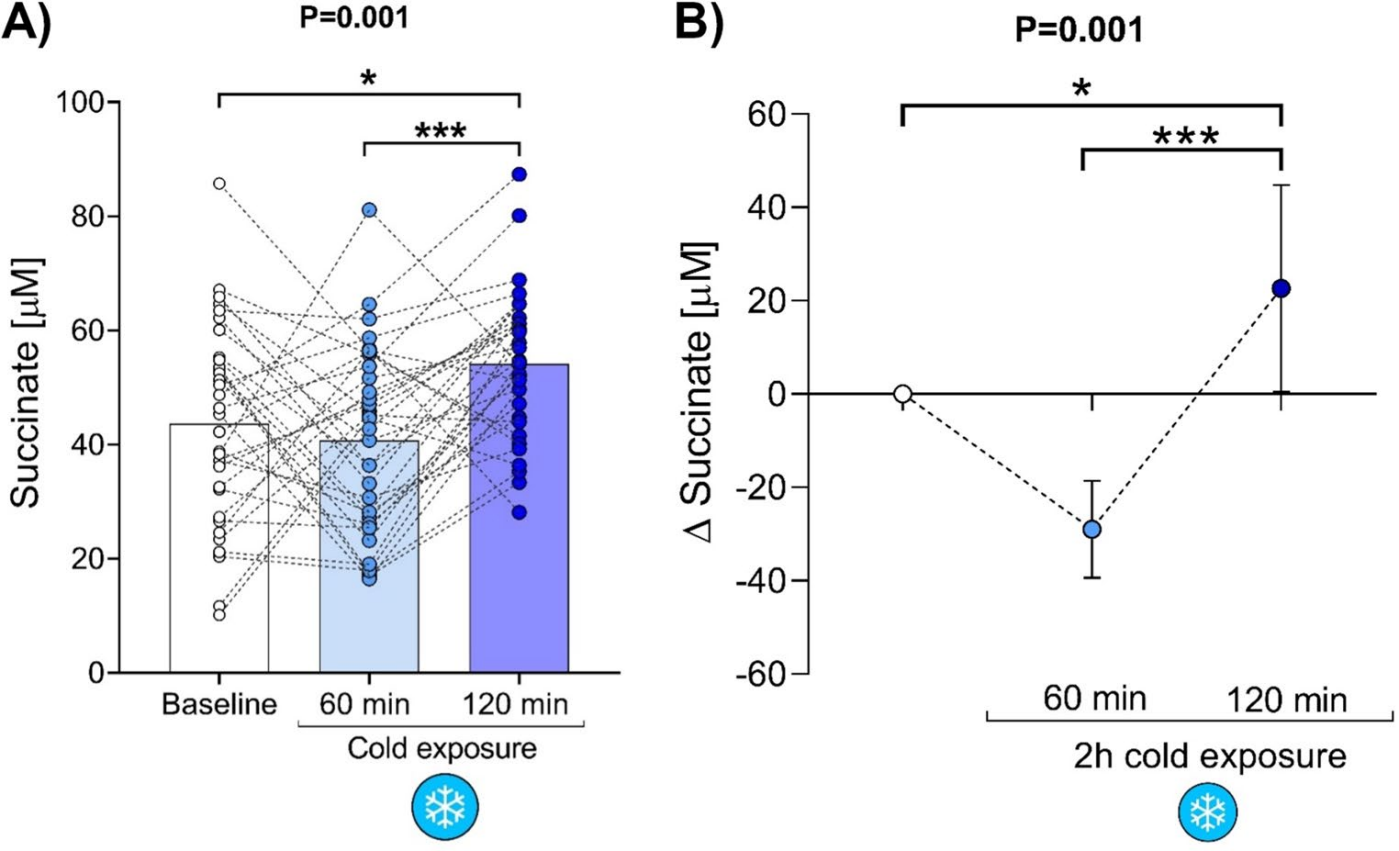
Plasma succinate levels during a 2-h personalized cooling protocol in young adults (*n* = 33). (**A**) Individual changes in plasma succiante levels where data is presented as means (bars) and individual values (circles). (**B**) Changes at 60 and 120 min in plasma susccinate vs. baseline levels, where data is presented as mean (circle) and standad deviation (error bars). P values were obtained from linear mixed-effects models (LMM) with time as the fixed factor and random effects for subjects. Post hoc analyses were performed using Bonferroni correction for multiple comparisons. *P < 0.05; ***P < 0.001

Circulating succinate levels changed significantly over time in response to cold exposure (*P* = 0.001). Mean concentrations were 43.6 ± 17.6 µM at baseline, 40.7 ± 16.4 µM at 60 min (*P* = 0.985 vs. baseline), and 54.1 ± 13.0 µM at 120 min (+ 23.8%, *P* = 0.017 vs. baseline; *P* < 0.001 vs. 60 min). Correspondingly, the mean change (Δ) from baseline was − 2.9 ± 22.6 µM at 60 min and + 10.5 ± 22.2 µM at 120 min, respectively. These results remained unchanged after adjustment for cooling vest temperature, body surface area, and PET-CT acquisition timing, indicating that the observed time-dependent changes were independent of these factors (all *P* < 0.05). These dynamic changes were accompanied by substantial interindividual variability, as evidenced by the mean individual percent changes (17 ± 90% at 60 min; 56 ± 108% at 120 min). Given the variability in both baseline plasma succinate concentrations and the magnitude and direction of change upon cold exposure (Fig. 1), we investigated potential predictors of the succinate response using stratification-based approaches and multivariable regression analyses. Stratification by sex, BMI, LBM, and VAT mass did not reveal any significant determinant factor influencing succinate response (Fig. S1 A-D; all P_interaction_≥0.202).

**Fig. 2:**
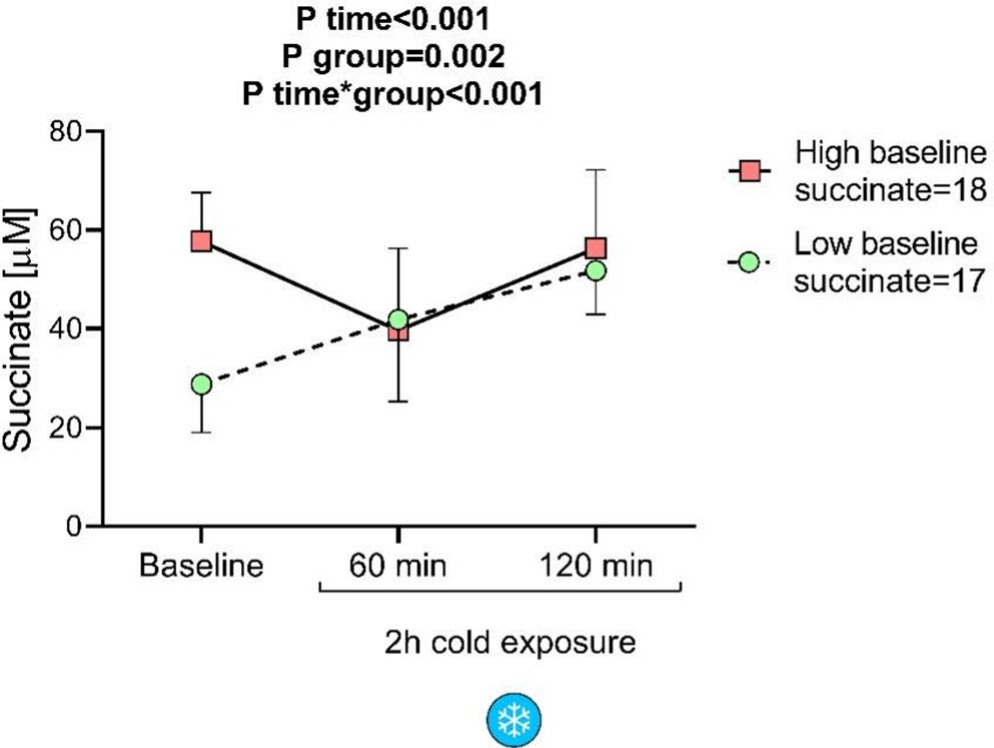
Changes on plasma succinate levels during a 2-h personalized cooling protocol in young adults (*n* = 33), stratified by baseline succinate levels. Data are presented as means by green circles for the low baseline succinate group (*n* = 16; 10.1–42.2µM baseline succinate range) or red squares for the high baseline succinate group (*n* = 17; 45.3–85,6µM baseline succinate range) and standard deviations (error bands). P values were obtained from linear mixed-effects models (LMM) including fixed effects for time and group, and random effects for subjects

Next, we performed stepwise linear regression analyses incorporating a comprehensive panel of phenotypic variables, grouped into four predefined categories (i.e., body composition, cardiometabolic markers, physical fitness, and BAT-related outcomes), as detailed in Table 1. No significant predictors of the succinate response were identified in any model (all *P* > 0.05). We also tested models using succinate levels at 60–120 min as outcomes and baseline levels as predictors, but these too yielded no significant associations (all *P* > 0.05). Given the absence of significant predictors of cold-induced changes in succinate levels, and the clear interindividual variability observed in Fig. 1 (with participants exhibiting either an increase or a decrease in circulating succinate) we conducted subgroup, descriptive and hypothesis-generating analyses by stratifying individuals by median baseline levels (low: 10.1–42.2 µM; high: 45.3–85.7 µM) to explore potential differential response patterns (Table S1). This stratification revealed a significant *Group x Time* interaction (*P* < 0.001), distinguishing two different groups with divergent response patterns (Fig. 2). Compared to baseline, individuals in the low-baseline group exhibited a + 46.0% increase in plasma succinate from 28.7 ± 9.7µM to 41.9 ± 16.6 µM at 60 min; (Δ = 13.1 ± 16.7 µM; *P* = 0.021) and a + 80.5% increase to 51.8 µM at 120 min (Δ = 25.0 ± 14.0 µM; *P* < 0.001) (Fig. 2). In contrast, individuals in the high-baseline group exhibited a − 31.4% reduction in plasma succinate from 57.7 ± 10.0 µM at baseline to 39.6 ± 16.6 µM at 60 min (Δ=–18.0 ± 16.1 µM; *P* < 0.001), followed by a − 2.4% reduction toward baseline levels to 56.3 ± 16.0 µM at 120 min (Δ=–1.4 ± 22.1 µM; *P* = 0.003) (Fig. 2). These results persisted after adjusting separately for cooling vest temperature, body surface area, and PET-CT date and time of the day. This divergent succinate response was not attributable to phenotypic differences between the low and high baseline succinate groups (Table S1).

### Changes in plasma succinate levels during cold exposure are associated with brown adipose tissue radiodensity in participants with low baseline succinate

**Fig. 3:**
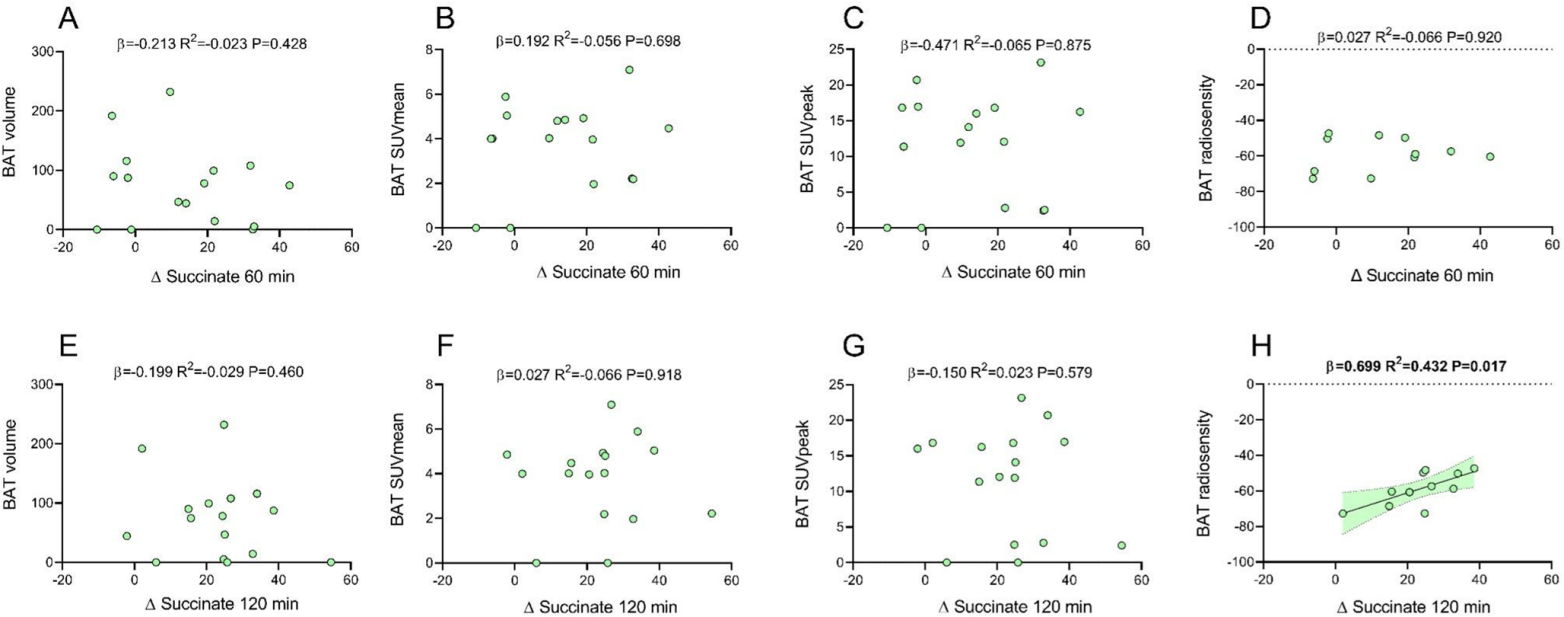
Linear regression analyses between changes in succinate levels during cold exposure and brown adipose tissue (BAT) parameters in individuals with low baseline succinate levels. Panel **A**) Δ succinate at 60 min (Δ = change from baseline) versus BAT volume (*n* = 16); Panel **B**) Δ succinate at 60 min versus BAT standardized uptake value (SUV) mean (*n* = 16); Panel **C**) Δ succinate at 60 min versus BAT SUV peak (*n* = 16); Panel **D**) Δ succinate at 60 min versus BAT radiodensity (*n* = 11); Panel **E**) Δ succinate at 120 min versus BAT volume (*n* = 16); Panel **F**) Δ succinate at 120 min versus BAT SUV mean (*n* = 16); Panel **G**) Δ succinate at 120 min versus BAT SUV peak (*n* = 16); Panel **H**) Δ succinate at 120 min versus BAT radiodensity (*n* = 11). All panels show standardized beta coefficients, adjusted R² values, and P-values derived from simple linear regression analyses

We first performed linear regression analyses across the entire cohort to assess the relationship between changes in plasma succinate concentrations (Δ succinate 60 and 120 min) and BAT parameters, including volume, ^18^F-FDG uptake, and radiodensity. No significant associations were detected when considering all individuals together (Fig. S2A–H; all *P* ≥ 0.333). However, a distinct pattern emerged when participants were stratified based on their baseline plasma succinate levels. Among individuals with low baseline succinate levels, changes in succinate concentrations at 120 min, but not 60 min, were positively associated with BAT radiodensity (β = 0.699, R²=0.432, *P* = 0.017; Fig. 3H). No other significant associations were observed within the low baseline group (Fig. 3A–G; all *P* ≥ 0.309), nor were any associations detected in individuals with high baseline succinate levels (Fig. S3 A–H; all *P* ≥ 0.321). These associations remained consistent after adjustment for cooling vest temperature, body surface area, and PET-CT date and time of day (all *P* < 0.05).

## Discussion

Our study shows that changes in succinate levels during a 2-h personalized cold exposure protocol in humans are heterogeneous and exhibit limited associations with BAT-related outcomes. No significant associations between cold-induced succinate changes and BAT volume or ^18^F-FDG uptake were observed in the full sample. Exploratory subgroup analyses identified a positive association between Δ succinate at 120 min and BAT radiodensity in individuals with low baseline succinate levels; however, this finding was restricted to a single BAT-related parameter and should be interpreted cautiously. Importantly, the absence of a thermoneutral control condition precludes definitive attribution of the observed succinate changes specifically to cold exposure. Therefore, our findings support the existence of heterogeneous systemic succinate responses during our experimental protocol but do not provide evidence for a relationship between circulating succinate dynamics and BAT activity in humans.

Non-shivering thermogenesis is a vital survival mechanism in endothermic mammals. It is influenced by body size, with smaller mammals generally exhibiting greater thermogenesis [25]. This heat-generating process depends in BAT primarily relies on UCP1, which dissipates energy as heat through fatty acid oxidation [26]. Although other UCP1-independent thermogenic mechanisms have also been described [3, 4], there is currently no direct evidence linking these pathways to the physiological activation or metabolic function of BAT in humans. Mills et al. demonstrated that cold exposure increases systemic succinate levels in mice, triggering BAT thermogenesis [13]. Accordingly, we observed a significant increase in circulating succinate levels following acute cold exposure, supporting the idea that succinate may act as a metabolic signal during thermogenic activation. However, this response was heterogeneous and not observed in all subjects, showing interindividual variability in succinate levels production and/or clearance. Regarding succinate production, the cooling protocol of Mills et al. (4 °C for 4 h) induced visual shivering and skeletal muscle contractions in mice, which contributed to circulating succinate via glycolysis and TCA cycle turnover [13]. In contrast, our study was specifically designed to minimize shivering [18] and therefore, the expected flux of succinate from contracting skeletal muscle should be low. Nonetheless, low-level involuntary muscle activity may still occur below the threshold of visual detection (as reported by electromyography (EMG) [27]), and therefore, we cannot rule out that muscle may have contributed to succinate production through contractile mechanisms like those triggered by low-intensity exercise [15, 28]. Because succinate is a central intermediate of mitochondrial metabolism, multiple tissues could potentially contribute to circulating succinate dynamics during cold exposure. However, the present study did not directly assess tissue-specific succinate production, uptake, or mitochondrial metabolism. Therefore, potential contributions from skeletal muscle, liver, white adipose tissue, or BAT remain speculative and require direct experimental investigation.

Despite we expected a uniform increase in circulating succinate in response to cold exposure, our findings revealed substantial interindividual variability. Due to the uneven sex distribution in our cohort, analyses were stratified by sex and so did by other factors known to influence circulating succinate levels, such as BMI [29] and VAT mass [30], and LBM [13] as a potential primary source of succinate in response to cold exposure. Notably, individuals in the low- and high-baseline succinate groups did not differ in body composition, cardiometabolic markers, physical fitness, or BAT parameters.), suggesting that the divergent succinate responses likely reflect heterogeneous metabolic adaptations to cold, independent of these phenotypic variables [31]. Thus, in individuals with low baseline succinate In addition, genetic polymorphisms affecting enzymes involved in succinate metabolism, mitochondrial, the steady increase in plasma succinate during cold exposure could reflect enhanced production or mobilization from metabolically active tissues such as skeletal muscle or liver [17, 32, 33]. Skeletal muscle, in particular, remains actively engaged during prolonged cold exposure through subtle contractions, potentially contributing to succinate release [15, 16]. Conversely, individuals with high baseline succinate levels exhibited an initial decrease in plasma succinate at 60 min. This observation is temporally compatible with previous murine studies reporting rapid BAT succinate uptake during cold exposure [13], although such mechanisms were not assessed here. The return to baseline succinate levels by 120 min may reflect compensatory physiological responses, such as increased succinate production or redistribution, that restore homeostasis and sustain thermogenesis. The delayed increase in circulating succinate observed after prolonged cold exposure may indicate that systemic succinate dynamics do not fully parallel the rapid BAT activation described in previous thermographic studies, although this interpretation remains speculative [34]. Rather than acting as an immediate trigger of thermogenesis, circulating succinate may reflect a secondary, integrated metabolic response to sustained cold exposure in humans. Furthermore, cold stress enhances hepatic gluconeogenesis, which involves TCA cycle intermediates and may alter TCA flux. Depending on substrate availability and anaplerotic inputs, this process could modulate succinate levels [32, 35]. Importantly, within the context of our study, all participants underwent a minimum of 6 h of fasting prior to cold exposure, a condition that minimizes acute postprandial fluctuations in circulating succinate. Consistent with our previous observations showing that succinate transiently increases following nutrient intake but returns to baseline within ~ 180 min [20], this standardized fasting state reduces the likelihood that the observed changes are driven by recent food intake. In addition, these patterns remained unchanged after adjustment for PET-CT acquisition time and related covariates, arguing against a major contribution of temporal or fasting-related effects to the observed succinate dynamics.

Although succinate has been shown to directly support thermogenesis in murine models [13], our data do not provide evidence for a comparable relationship in humans at the systemic level. One possible explanation for the lack of associations is that ^18^F-FDG PET/CT primarily reflects glucose uptake and may not fully capture other aspects of BAT oxidative metabolism [36, 37]. Interindividual differences in sympathetic tone, tissue perfusion, and glucose clearance might further confound ^18^F-FDG–based assessments, particularly in modestly sized cohorts [38, 39]. In an explofator analsis, among individuals with low baselinelevels, the cold-induced succinate chanes at 120 min was positively associated with BAT radiodensity, a marker of BAT intracellular triglyceride content [40]. Indeed, FDG uptake and CT-derived radiodensity reflect distinct biological dimensions of BAT physiology, including glucose utilization versus tissue composition and lipid content, and are not expected to be tightly coupled under all conditions. In line with this, we previously have reported discordant patterns, including higher FDG uptake despite lower radiodensity across groups [41]. This radiological feature (BAT radiodensity, measured in HU by CT) is determined by the relative amounts of lipid, water, and vascular content, as well as the cellular architecture of the tissue, such as multilocular brown adipocytes versus unilocular white adipocytes [40, 42, 43]. Importantly, BAT radiodensity was measured only once at 120 min, and no within-subject changes were evaluated. Therefore, our data do not allow us to relate changes in circulating succinate to changes in BAT radiodensity over time. Within this context, because acute BAT activation during cold exposure leads to intracellular lipid mobilization, increased perfusion, and hydration, these changes can result in higher radiodensity values after cold exposure [24, 40]. Consistently, cold-stimulated BAT regions with higher HU have been shown take up more circulating non-esterified fatty acids (NEFAs) than regions with lower HU [40]. In this framework, preclinical studies suggest that succinate can modulate oxidative metabolism in murine BAT under certain experimental conditions [13], although this possibility was not directly assessed in the present study. Supporting this interpretation, preclinical studies have shown that exogenous succinate can enhance oxidative metabolism in murine BAT, and promote thermogenic activation [13, 44, 45]. Nevertheless, our data do not directly assess mitochondrial function or lipid turnover in BAT, and the observed association is restricted to a specific subgroup and a single BAT-related parameter. While our study focused on circulating succinate as a candidate thermogenic metabolite, cold exposure is known to modulate a broader range of circulating metabolites [46], and future studies incorporating comprehensive metabolomic profiling will be necessary to determine how succinate integrates within this wider metabolic network and to identify potential metabolites associated with BAT activation in humans. Importantly, because this association emerged from exploratory subgroup analyses involving modest sample sizes and multiple statistical comparisons, the possibility of false-positive findings cannot be excluded. Whether circulating succinate contributes to BAT oxidative metabolism in humans remains unknown and was not addressed by the present study. Additionally, BAT activity was assessed using ^18^F-FDG PET/CT, which primarily reflects glucose uptake and may not fully capture substrate-specific oxidative metabolism or thermogenic activity.

## Limitations

Although circulating succinate increased after prolonged cold exposure and this effect was independent of time-of-day and related covariates, the absence of a thermoneutral time-matched control condition precludes definitive attribution of this effect exclusively to cold. Therefore, potential contributions from temporal, fasting-related, or other physiological fluctuations cannot be completely excluded. Nevertheless, within-subject cold exposure paradigms without thermoneutral PET-CT control conditions are common in human BAT imaging studies due to logistical complexity, participant burden, and radiation exposure associated with repeated PET-CT acquisitions. The observational design precludes causal inference and the relatively small sample size, particularly for subgroup analyses and BAT radiodensity measurements, limits statistical power and may have reduced the ability to detect weaker associations between circulating succinate dynamics and BAT-related outcomes. Consequently, both positive and negative findings should be interpreted cautiously. Third, the use of a static PET/CT scan can provide only a unique snapshot at 120 min, limiting the ability to capture real-time dynamic changes in FDG and radiodensity and circulating succinate [47]. Another limitation is the reliance on ¹⁸F-FDG PET to assess BAT activity; although widely used, FDG uptake reflects general glucose uptake rather than tissue-specific metabolic functions or thermogenesis [48]. Future studies should combine dynamic CT radiodensity measurements, alternative PET tracers like ¹¹C-acetate and ^15^O_2_, and circulating metabolite profiling to better characterize BAT metabolism in humans [49], ideally incorporating matched thermoneutral conditions to disentangle cold-specific from temporal effects. Consequently, our findings should be interpreted as physiological associations observed during acute cold exposure rather than definitive evidence of cold-specific causal effects.

## Conclusion

In conclusion, acute cold exposure was associated with heterogeneous changes in circulating succinate levels in humans, with substantial interindividual variability and limited associations with BAT PET/CT-derived outcomes. Under the present experimental conditions, these findings do not support circulating succinate dynamics as a robust marker of BAT activity in humans.

## Supplementary Information

Below is the link to the electronic supplementary material.

Supplementary Material 1 (DOCX 64.0 KB)

Supplementary Material 2 (DOCX 636 KB)

## Data Availability

No datasets were generated or analysed during the current study.

## Declarations

### Data sharing

All relevant data is included in the article; further enquiries can be directed to the corresponding authors.

### Database and data handling procedures

The study has a data management plan that strictly follows the regulations of the University of Granada, which ensures data protection all the project. The data are treated according to EU regulation 2016/679 (e.g., pseudo-anonymization, security measures to prevent unauthorized access) and national legislation (Seventeenth additional provision, Health data treatments) of Spanish Organic Law 3/2018, of December 5th, on the Protection of Personal Data and guarantee of digital rights. All the data and research material are stored following the guidelines of the University of Granada and archived in a Central biobank. Our data management plan also follows the H2020 FAIR principle, as we are making our research data accessible, interoperable, and reusable.
