# Supplementary material for: Circulating succinate changes during acute cold exposure are not related with brown adipose tissue in humans
Source: J Physiol Biochem. 2026 Aug 1;82(1):76. doi: 10.1007/s13105-026-01212-z (PMC13428776; doi:10.1007/s13105-026-01212-z)
Supplement: Supplementary file 1 — Supplementary Material 1 (DOCX 64.0 KB) [file 13105_2026_1212_MOESM1_ESM.docx]

**SUPPLEMENTARY MATERIAL**

***Cardiometabolic risk factors***

Serum levels of traditional cardiometabolic risk factors were determined using standard laboratory techniques. Glucose concentrations were quantified on an AU5832 analyser (Beckman Coulter, Brea, CA, USA) using Beckman Coulter reagent (OSR6521). Insulin levels were measured via chemiluminescence immunoassay on the UniCel DxI 800 analyser (Beckman Coulter), employing Beckman Coulter chemiluminescent reagents (33410). Total cholesterol (TC), high-density lipoprotein cholesterol (HDL-C), triglycerides (TG), apolipoproteins A and B, and liver enzymes such as glutamic pyruvic transaminase (GPT), gamma-glutamyl transferase (GGT), and alkaline phosphatase (ALP), along with creatinine and creatine kinase, were analysed using the AU5832 spectrophotometer with corresponding Beckman Coulter reagents (OSR6116, OSR60118, OSR6187, 446410, 447730, OSR6507, OSR6520, OSR6204, and OSR6678). Low-density lipoprotein cholesterol (LDL-C) was calculated using the Friedewald formula: [TC (mM) – HDL-C (mM) - 0.45 × TG (mM)]. C-reactive protein (CRP) levels were measured by immunoturbidimetric assay (OSR6299) using the AU5832 spectrophotometer. Insulin sensitivity was estimated using the homeostasis model assessment of insulin resistance (HOMA-IR) index [1]. Leptin and adiponectin were determined using the Human Adipokine Magnetic Bead Panel (#HADK2MAG-61K, HADK1MAG-61K; Luminex).

***Physical fitness parameters***

The cardiorespiratory fitness assessment involved a maximum effort test conducted on a treadmill (Pulsar treadmill, H/P/Cosmos Sports & Medical GmbH, Nussdorf-Traunstein, Germany) using a modified Balke protocol [2]. The session commenced with participants walking at a pace of 3 km/h for 1 minute, followed by 2 min at 4 km/h, both performed on a level treadmill (0% incline) to facilitate warm-up [2]. The test began with participants walking at 5.3 km/h on a flat treadmill (0% incline). Subsequently, the treadmill incline was increased by 1% every minute, continuing until participants reached volitional exhaustion [2]. At this point, individuals started a 5 min recovery walking at 4 km/h and 0% grade [2]. Following the test, participants engaged in a 5-minute recovery period, walking at 4 km/h on a flat treadmill (0% incline). Throughout the entire test, participants were equipped with a heart rate monitor (Polar RS800CX, Polar Electro Öy, Kempele, Finland), 10 electrodes for electrocardiogram monitoring, and a Hans-Rudolph plastic mask (model 7400, Hans Rudolph Inc., Kansas City, MO, USA) connected to a preVent™ metabolic flow sensor (Medical Graphics Corp, St Paul, MN, USA). This setup was used for respiratory gas exchange analysis with a CPX Ultima CardioO2 gas exchange system (Medical Graphics Corp, St Paul, MN, USA). Oxygen consumption (VO_2_) and carbon dioxide production (VCO_2_) were recorded during the test, and the peak VO_2_ (VO_2_peak) was determined as the highest value obtained, after artifact correction if necessary.

The muscle strength assessment involved a combination of four tests: i) a maximum isometric strength test using the leg press, ii) a maximum isometric handgrip strength test, and iii) and iv) two 1-repetition maximum (1-RM) tests with the bench press and leg press, respectively [3]. Participants first performed the maximum isometric strength test with the leg press. They were positioned in the A300 Leg Press machine (Model 2531, Keiser Corporation, Fresno, CA, USA) and instructed to complete two 3-second repetitions, with a 2-minute rest between sets. During each repetition, participants were encouraged to push as forcefully as possible throughout the entire duration. Next, participants completed the handgrip strength test, performing two repetitions with each hand, with a 1-minute rest in between, using a Takei 5401 digital Grip-D hand dynamometer (Takei, Tokyo, Japan) [4]. For this test, participants remained in a standing position, with the exercising arm parallel to and slightly separated from the trunk. They were instructed to gradually and continuously squeeze the grip as hard as possible. For men, the grip span of the dynamometer was fixed at 5.5 cm, while for women, it was adjusted according to hand size, following a validated equation [4]. The highest strength value recorded for each hand was selected, and the average of both hands was used for subsequent analysis.

Individuals then performed the leg press 1-RM test using the same leg press machine. After completing one set of 10 repetitions with a self-selected lightweight for warm-up, they were instructed to perform one set of 8 repetitions, selecting a resistance with which they could complete at least 15 repetitions. After a 1-minute recovery, the resistance load was increased by the study personnel to a level that would allow for fewer than 10 repetitions, and participants were instructed to perform as many repetitions as possible. If they were able to perform more than 10 repetitions, they rested for 5 min before repeating the test with a higher load. A maximum of three attempts were allowed to assess the 1-RM (with a set of fewer than 10 repetitions). Lastly, participants performed the bench press 1-RM test following the same procedure, using a bench within a pneumatic power rack (Power rack, Model 3111, Keiser Corporation, Fresno, CA, USA). The 1-RM for both exercises was estimated using the Wathen equation mentioned [5].

**SUPPLEMENTARY MATERIAL REFERENCES**

1. Matthews JC. Instability of brain synaptosomal membrane preparations to repeated ultracentrifugation in isoosmotic density gradients. Life Sci. 1985;37:2467–73.

2. BALKE B, WARE RW. An experimental study of physical fitness of Air Force personnel. U S Armed Forces Med J [Internet]. 1959;10:675–88. Available from: http://www.ncbi.nlm.nih.gov/pubmed/13659732

3. Martinez-Tellez B, Sanchez-Delgado G, Amaro-Gahete FJ, Acosta FM, Ruiz JR. Relationships between cardiorespiratory fitness/muscular strength and 18F-fluorodeoxyglucose uptake in brown adipose tissue after exposure to cold in young, sedentary adults. Sci Rep. 2019;9:1–9.

4. Ruiz-Ruiz J, Mesa JLM, Gutiérrez A, Castillo MJ. Hand size influences optimal grip span in women but not in men. Journal of Hand Surgery. 2002;27:897–901.

5. Phillips N. Essentials of Strength Training and Conditioning. Physiotherapy. 1997;83:47.
