# Supplementary material for: Circulating succinate changes during acute cold exposure are not related with brown adipose tissue in humans
Source: J Physiol Biochem. 2026 Aug 1;82(1):76. doi: 10.1007/s13105-026-01212-z (PMC13428776; doi:10.1007/s13105-026-01212-z)
Supplement: Supplementary file 2 — Supplementary Material 2 (DOCX 636 KB) [file 13105_2026_1212_MOESM2_ESM.docx]

**Table S1**. Characteristics of the participants with low (n = 16) and high-baseline (n = 17) succinate levels.

|  | Low succinate  (10.1–42.2µM) | | High succinate  (45.3–85.6µM) | | P  group |
| --- | --- | --- | --- | --- | --- |
|  | Mean | SD | Mean | SD |  |
| Age (years) | 21.8 | 2.4 | 21.8 | 2.1 | 0.994 |
| Sex (male/female) | 6/10 | | 7/10 | | 0.829 |
| *Body composition* |  |  |  |  |  |
| BMI (kg/m^2^) | 24.2 | 5.7 | 23.7 | 5.1 | 0.797 |
| Lean mass index (kg/m^2^) | 14.8 | 2.5 | 14.5 | 2.4 | 0.717 |
| Fat mass index (kg/m^2^) | 8.2 | 3.5 | 7.9 | 3.3 | 0.826 |
| Fat mass (%) | 33.3 | 7.2 | 33.2 | 8.3 | 0.956 |
| VAT mass (g) | 309.3 | 205.5 | 297.1 | 195.8 | 0.862 |
| *Cardiometabolic risk factors* |  |  |  |  |  |
| Glucose (mg/dL) | 88.5 | 8.9 | 86.9 | 5.7 | 0.557 |
| Insulin (µIU/mL) | 10.1 | 10.8 | 8.4 | 3.8 | 0.551 |
| HOMA-IR | 2.4 | 3.1 | 1.8 | 0.8 | 0.460 |
| Total cholesterol (mg/dL) | 169.2 | 45.2 | 158.4 | 22.1 | 0.399 |
| HDL-C (mg/dL) | 52.6 | 11.4 | 50.2 | 10.9 | 0.541 |
| LDL-C (mg/dL) | 99.9 | 33.3 | 91.4 | 17.8 | 0.378 |
| Triglycerides (mg/dL) | 88.4 | 70.9 | 84.0 | 38.6 | 0.830 |
| APOA1 (mg/dL) | 142.5 | 30.8 | 144.9 | 23.3 | 0.832 |
| APOB (mg/dL) | 62.8 | 16.7 | 65.1 | 14.4 | 0.729 |
| Adiponectin (mg/L) | 12.8 | 11.2 | 10.9 | 5.7 | 0.561 |
| Leptin (µg/L) | 4.6 | 3.1 | 4.2 | 2.6 | 0.686 |
| GPT (IU/L) | 25.8 | 32.8 | 18.6 | 12.4 | 0.419 |
| GGT (IU/L) | 27.1 | 36.5 | 19.9 | 12.0 | 0.460 |
| ALP (IU/L) | 74.0 | 29.8 | 72.1 | 18.2 | 0.831 |
| Creatinine (mg/dL) | 0.8 | 0.1 | 0.9 | 0.2 | 0.171 |
| Creatine kinase (μmol/L) | 144.6 | 185.2 | 109.3 | 49.3 | 0.466 |
| C-reactive protein (mg/L) | 2.4 | 2.2 | 2.4 | 3.3 | 0.960 |
| *Physical fitness* |  |  |  |  |  |
| Handgrip strength (kg) | 31.1 | 7.6 | 31.0 | 9.1 | 0.974 |
| RM bench press (kg) | 33.7 | 13.2 | 33.2 | 18.0 | 0.928 |
| RM leg press (kg) | 223.7 | 77.3 | 197.2 | 68.1 | 0.303 |
| VO_2_peak (mL/kg/min) | 39.7 | 7.1 | 45.3 | 8.4 | 0.055 |
| *Brown adipose tissue* |  |  |  |  |  |
| BAT volume (mL) | 76.4 | 98.1 | 55.8 | 52.2 | 0.658 |
| BAT SUVmean | 4.0 | 2.7 | 3.7 | 2.2 | 0.803 |
| BAT SUVpeak | 13.1 | 14.2 | 8.6 | 12.1 | 0.989 |
| BAT radiodensity (HU) | -58.8 | 18.8 | -58.1 | 6.9 | 0.818 |

Data presented as mean and standard deviation (SD), otherwise stated. BAT radiodensity sample size: *Abbreviations*: ALP, alkaline phosphatase; APOA1, apolipoprotein A1; APOB, apolipoprotein B; BAT, brown adipose tissue; BMI, body mass index; GGT, gamma-glutamyl transferase; GPP, glutamic pyruvic transaminase; HDL-C, high-density lipoprotein cholesterol; HOMA-IR, homeostatic model assessment of insulin resistance index; HU, Hounsfield Units; LDL-C, low-density lipoprotein cholesterol; RM: repetition maximum; SUV, standardized uptake value; VAT, visceral adipose tissue: VO_2_: oxygen consumption.

**
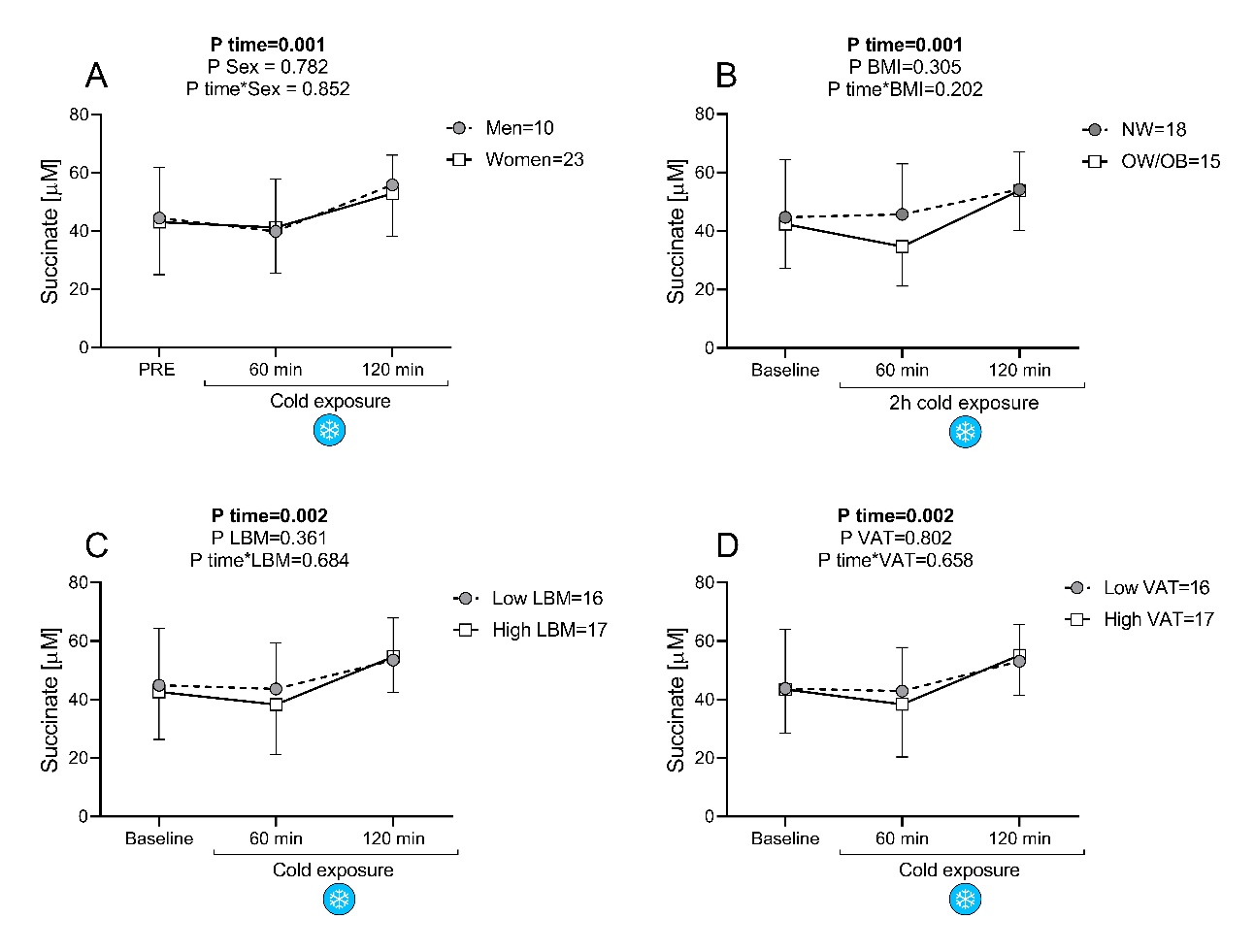
**

**Figure S1. Changes in plasma succinate levels during a 2-h personalized cooling protocol in young adults**. Panel A: Plasma succinate levels before and after cold exposure, stratified by sex into men (n = 10) and women (n = 23). Panel B: stratifying for body weight status into individuals with normal-weight (n = 18) and with overweight/obesity (n = 15). Panel C: stratifying for lean body mass into low (n = 16) and high (n = 17). Panel D: Plasma succinate levels before stratifying for visceral adipose tissue mass into low (n = 16) and high (n = 17). Data are presented as means (circles or squares) and standard deviations (error bands). P values were obtained from linear mixed-effects models (LMM) including fixed effects for time and group, and random effects for subjects. *Abbreviations:* BAT, brown adipose tissue; LBM, lean body mass; NW, individuals with normal-weight; OW/OB, individuals with overweight/obesity.; VAT: visceral adipose tissue.


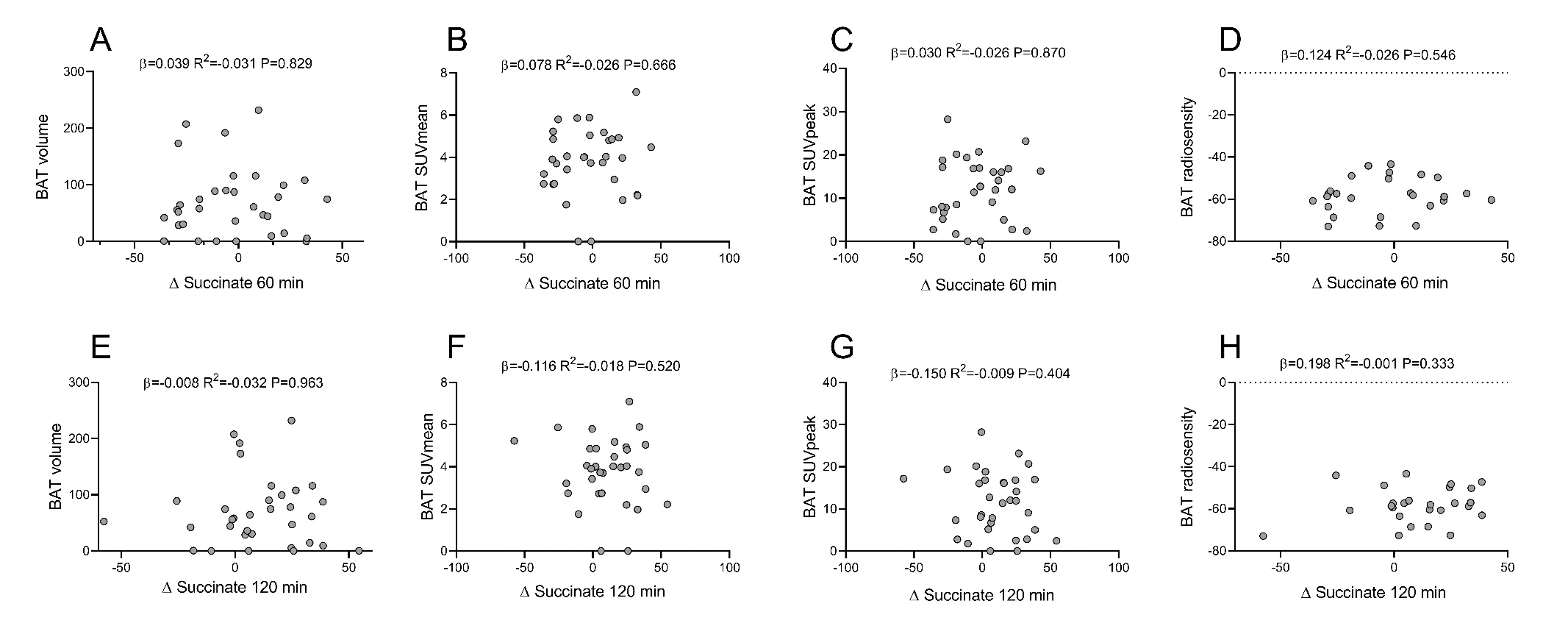


**Fig S2.** Linear regression analyses of the association between changes in plasma succinate levels during cold exposure and brown adipose tissue (BAT) parameters in young individuals (n = 33). Panel A) Δ succinate at 60 min (Δ = change from baseline) versus BAT volume (n = 33); Panel B) Δ succinate at 60 min versus BAT standardized uptake value (SUV) mean (n = 33); Panel C) Δ succinate at 60 min versus BAT SUV peak (n = 33); Panel D) Δ succinate at 60 min versus BAT radiodensity (n = 26). Panel E) Δ succinate at 120 min versus BAT volume (n = 33); Panel F) Δ succinate at 120 min versus BAT SUV mean (n = 33); Panel G) Δ succinate at 120 min versus BAT SUV peak (n = 33); Panel H) Δ succinate at 120 min versus BAT radiodensity (n = 26). All panels show standardized beta coefficients, adjusted R² values, and P-values derived from simple linear regression analyses.


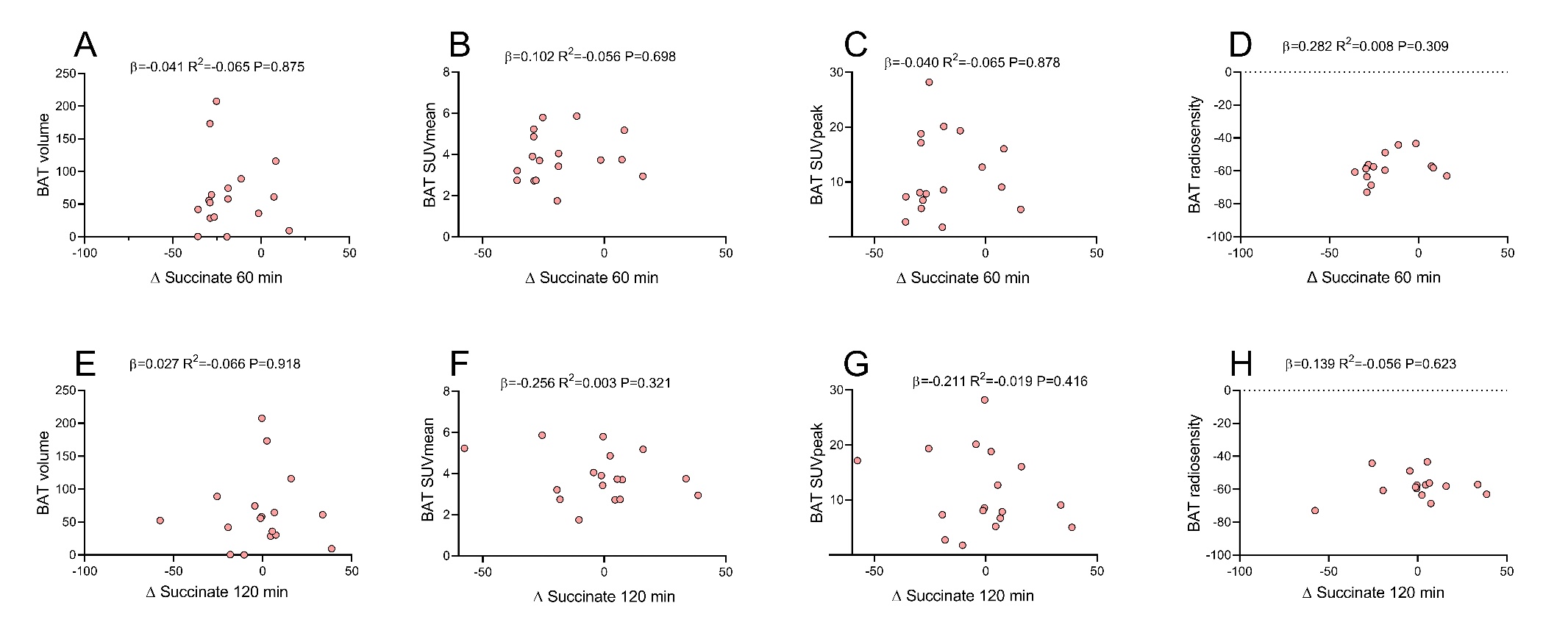


**Fig S3.** Linear regression analyses of the association between changes in plasma succinate levels during cold exposure and brown adipose tissue (BAT) parameters in individuals with high baseline succinate levels (n = 17). Panel A) Δ succinate at 60 min (Δ = change from baseline) versus BAT volume (n = 17); Panel B) Δ succinate at 60 min versus BAT standardized uptake value (SUV) mean (n = 17); Panel C) Δ succinate at 60 min versus BAT SUV peak (n = 17); Panel D) Δ succinate at 60 min versus BAT radiodensity (n = 15); Panel E) Δ succinate at 120 min versus BAT volume (n = 17); Panel F) Δ succinate at 120 min versus BAT SUV mean (n = 17); Panel G) Δ succinate at 120 min versus BAT SUV peak (n = 17); Panel H) Δ succinate at 120 min versus BAT radiodensity (n = 15). All panels show standardized beta coefficients, adjusted R² values, and P-values derived from simple linear regression analyses.
